# miR-181a-5p suppresses invasion and migration of HTR-8/SVneo cells by directly targeting *IGF2BP2*

**DOI:** 10.1038/s41419-017-0045-0

**Published:** 2018-01-16

**Authors:** Liang Wu, Wen-yan Song, Ya Xie, Lin-li Hu, Xiao-man Hou, Rui Wang, Yang Gao, Jing-na Zhang, Le Zhang, Wei-wei Li, Cheng Zhu, Zhi-ying Gao, Ying-pu Sun

**Affiliations:** 1 Reproductive Medical Center, the First Affiliated Hospital of Zhengzhou University, 450052 Zhengzhou, Henan China; 2 Henan Key Laboratory of Reproduction and Genetics, the First Affiliated Hospital of Zhengzhou University, 450052 Zhengzhou, Henan China; 3 Department of Gynecology and Obstetrics, the First Affiliated Hospital of Zhengzhou University, 450052 Zhengzhou, Henan China; 4 Department of Obstetrics and Gynecology, PLA General Hospital, 100853 Beijing, China; 5 State Key Laboratory of Stem Cell and Reproductive Biology, Institute of Zoology, Chinese Academy of Sciences, 100101 Beijing, China

## Abstract

Pre-eclampsia is a pregnancy-related disease that may cause maternal, neonatal and fetal morbidity and mortality and exists in 3–5% of pregnancies worldwide. The discovery of dysregulated microRNAs and their roles in placental development has provided a new avenue for elucidating the mechanism involved in this pregnancy-specific disorder. Here, the roles of human miR-181a-5p, a microRNA that is increased in both the plasma and placenta of severe pre-eclamptic patients, in invasion and migration of trophoblasts were investigated. Ectopic-expression of miR-181a-5p impaired the invasion and migration of HTR-8/SVneo cells, whereas miR-181a-5p inhibition had the opposite effects. *IGF2BP2*, which harbors a highly conserved miR-181a-5p-binding site within its 3ʹ-UTR, was identified to be directly inhibited by miR-181a-5p. Moreover, siRNAs targeting *IGF2BP2* imitated the effects of overexpressed miR-181a-5p on HTR-8/SVneo cell invasion and migration, whereas restoring *IGF2BP2* expression by overexpressing a plasmid encoding *IGF2BP2* partially reversed the studied inhibitory functions of miR-181a-5p. Thus, we demonstrated here that miR-181a-5p suppresses the invasion and migration of cytotrophoblasts, and its inhibitory effects were at least partially mediated by the suppression of *IGF2BP2* expression, thus shedding new light on the roles of miR-181a-5p in the pathogenesis of severe pre-eclampsia.

## Introduction

Normal proliferation/differentiation of human placental trophoblasts contributes to the proper function of the placenta. Dysregulated differentiation of trophoblast cells causes abnormal trophoblasts invasion and syncytialization and leads to pregnancy-related diseases including pre-eclampsia (PE)^1^. PE is a pregnancy-specific disease that may cause maternal and neonatal/fetal morbidities and mortalities, existing in 3–5% of pregnancies worldwide^2^. Although an imbalance of proangiogenic and antiangiogenic factors in circulation, including decreased placental growth factor (PlGF), as well as increased endoglin and fms-related tyrosine kinase 1 (FLT1) in soluble form, were implied to have a critical pathogenic role in PE^3^, the mechanisms involved remain largely unknown.

MicroRNA (miRNA), a set of non-coding small RNAs, plays regulatory roles by mainly inhibiting target function via directly interacting with its mRNA 3ʹ-untranslated region (3ʹ-UTR), with subsequently transcriptional degradation/translational repression^4^. Human miRNAs are highly expressed in the placenta^5^ and are substantially altered in the placenta from patients complicated with pregnancy-related diseases, such as PE^6–8^. MiRNAs in circulation have been suggested as promising biomarkers of pregnancy-related diseases, thus providing new diagnostic and therapeutic options during pregnancy^9^. In our previous work, significant increase of some plasma miRNAs including miR-181a-5p was found in circulation of patients with severe PE (sPE)^10^. Subsequently, the increase of plasma miR-181a-5p was confirmed in women with sPE^11^, as well as the elevation of placental miR-181a-5p in patients with sPE^7,8,12^. All these studies suggest the importance of miR-181a-5p in the pathogenesis of sPE. However, the molecular function of miR-181a-5p in placental development and its contributions to the development of sPE when deregulated have not been investigated.

The dominant theory suggests two main types of PE: placental PE and maternal PE, which are characterized by abnormalities originating from either a malfunctioning placenta or from environmental/maternal nutritional factors, respectively^13^. In the present study, we intended to discover the possible roles of miR-181a-5p in trophoblast invasion and migration. The elevation of placental miR-181a-5p was confirmed in severe pre-eclamptic placentas. Transwell assays were performed using trophoblast cells treated with mimic or inhibitor of miR-181a-5p. We further tested if insulin-like growth factor 2 mRNA-binding protein 2 (*IGF2BP2*) is a target directly inhibited by miR-181a-5p by using luciferase report assays. Combined with siRNA imitation assays and rescue experiments, we demonstrated that miR-181a-5p suppresses invasion and migration of trophoblasts at least partly by directly targeting *IGF2BP2*.

## Results

### miR-181a-5p is up-regulated in severe pre-eclamptic placentas, compared to normal placentas

**Fig. 1:**
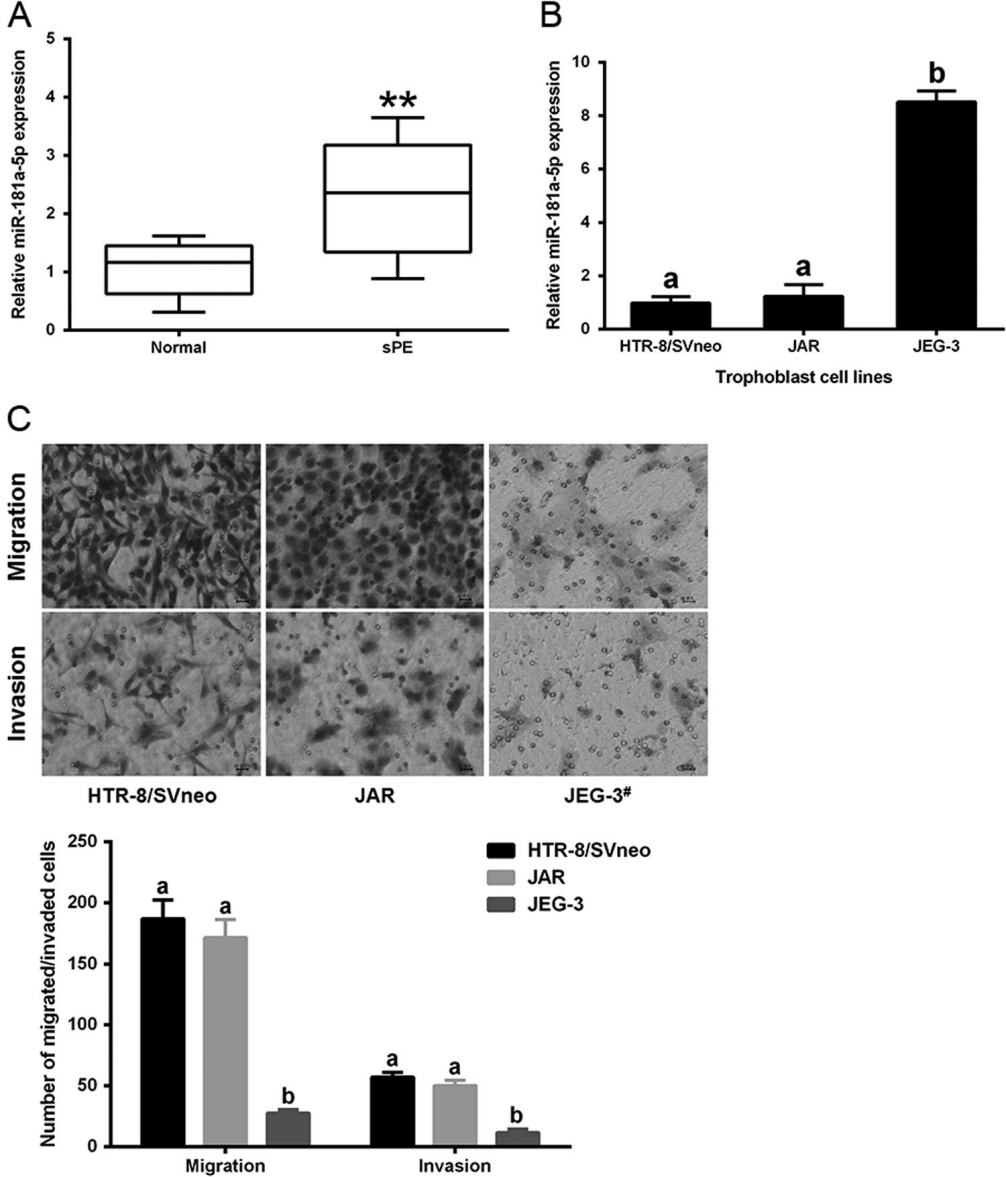
miR-181a-5p expression in human placentas and human trophoblast cells **a** Differential miR-181a-5p expression in severe pre-eclamptic placentas (*n* = 10) and normal placentas (*n* = 10) was assessed by qRT-PCR. **b** miR-181a-5p expression in three trophoblast cell lines, with the highest expression observed in JEG-3 cells and the lowest expression observed in HTR-8/SVneo cells. **c** Invasion and migration capacities of the three tested trophoblast cell lines. JEG-3 cells had significantly lower invasion/migration capacities than HTR-8/SVneo and JAR cells. Representative fields of invaded/migrated cells (at 200× original magnification, bar = 10 μm) are shown. ^#^The JEG-3 cells applied in invasion/migration assays were two-fold more than HTR-8/SVneo and JAR cells, as its weak invasion/migration capacities. The results are expressed as the mean ± SD based on at least three independent experiments. **P < 0.01; the values with diverse letters are also significantly different (*P* < 0.01)

Our previous report demonstrated the up-regulation of seven plasma miRNAs, including miR-181a-5p, in circulation of pre-eclamptic patients compared to that of normal pregnant females. In the beginning of this study, the up-regulation of miR-181a-5p was confirmed in severe pre-eclamptic placentas (Fig. 1a). We then examined miR-181a-5p expression of three trophoblasts lines: HTR-8/SVneo, JAR, and JEG-3 cells. MiR-181a-5p expression was top in JEG-3 cells and lowest in HTR-8/SVneo cells (Fig. 1b). Abnormal trophoblasts invasion/migration caused by dysregulated differentiation of trophoblast cells contributes to PE development. We subsequently tested the invasion/migration capacities of the above three trophoblasts lines and found that the JEG-3 cells, which had the highest miR-181a-5p expression, had the weakest invasion and migration abilities, about 10-fold lower than those of HTR-8/SVneo cells, which had the lowest miR-181a-5p expression; JAR cells had a slightly, but not significantly, lower capacities of invasion and migration compared to HTR-8/SVneo cells (Fig. 1c). These results suggested that the miR-181a-5p expression might be associated with trophoblast invasion and migration.

Since the invasion and migration capabilities of JEG-3 cells were too weak and the HTR-8/SVneo and JAR cells exhibited similar low miR-181a-5p expression and high invasion/migration capacities compared to JEG-3 cells, we chose HTR-8/SVneo, a cell line established by stable transfection of the gene encoding simian virus-40 (SV40) large T-antigen into normal first trimester human trophoblasts^14^, as the primary cell model to study the biological roles of miR-181a-5p in trophoblast invasion and migration.

### miR-181a-5p suppresses HTR-8/SVneo cell invasion and migration

**Fig. 2:**
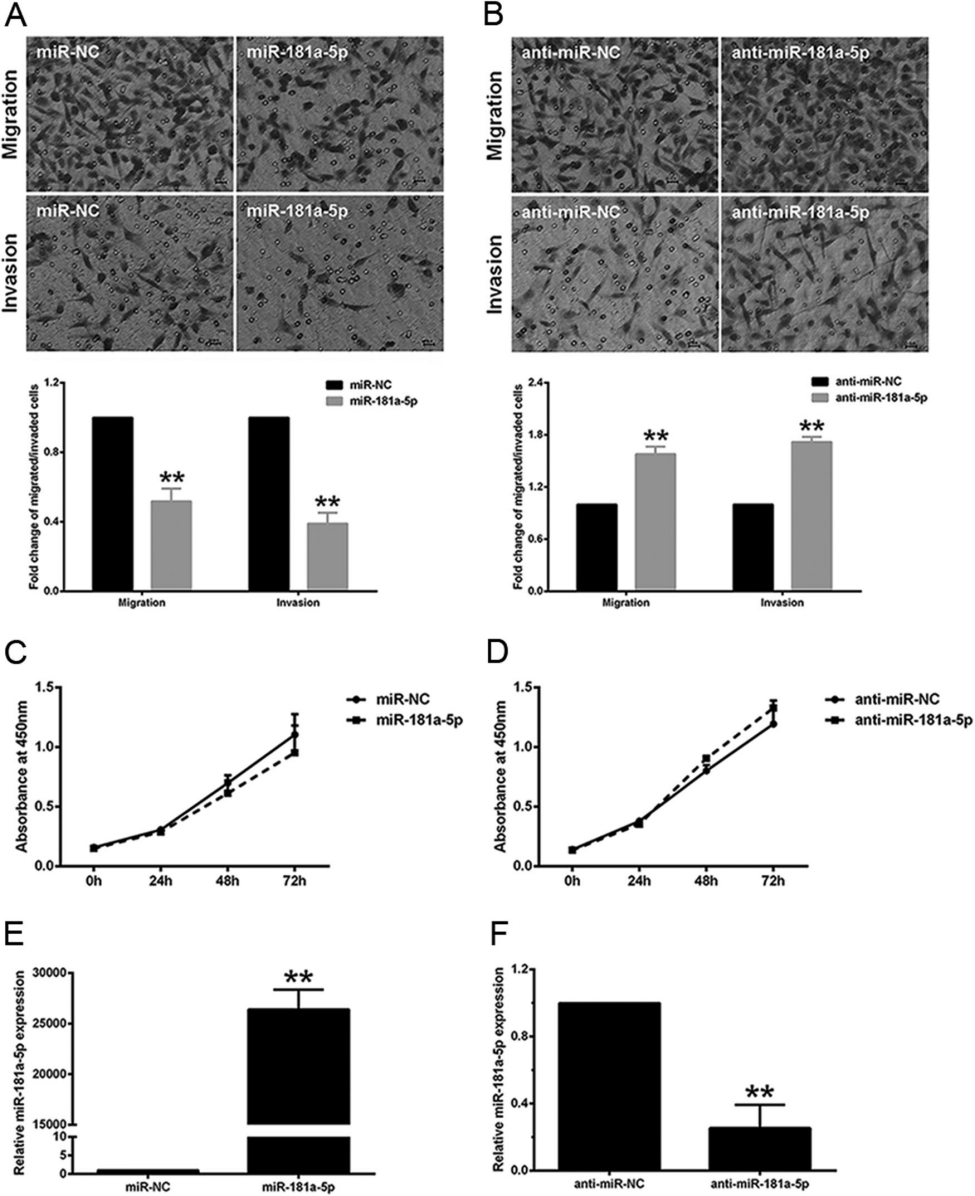
miR-181a-5p suppresses HTR-8/SVneo cell invasion and migration **a**, **b** HTR-8/SVneo cell invasion and migration were inhibited upon transfection of miR-181a-5p mimic **a**, and enhanced upon transfection of miR-181a-5p inhibitor **b**. Representative fields of invaded/migrated cells (at 200× original magnification, bar = 10 μm) are shown. **c**, **d** Transfected cells were subjected to CCK-8 assays in parallel with transwell assays in the presence/absence of Matrigel, and the changes in cell number at 0, 24, 48, and 72 h were measured. **e**, **f** Ectopic-expression and inhibition of miR-181a-5p were confirmed by qRT-PCR. The results are expressed as the mean ± SD based on at least three independent experiments. **P < 0.01

To find the possible roles of miR-181a-5p in invasion and migration of trophoblasts, we treated HTR-8/SVneo cells with either mimic or the corresponding negative control of miR-181a-5p, before using transwell assays with/without Matrigel to measure cell invasion/migration, respectively. The number of cells invading/migrating through transwell pores was significantly reduced after miR-181a-5p transfection (Fig. 2a). We subsequently assessed the effects of miR-181a-5p inhibition on HTR-8/SVneo cell invasion and migration by transfecting cells with the miR-181a-5p inhibitor. As expected, inhibiting miR-181a-5p significantly promoted HTR-8/SVneo cell invasion and migration compared to the corresponding negative control cells (Fig. 2b). Transfected cells were assigned to cell counting assays in 96-well plates to examine the change in cell number at 0, 24, 48, and 72 h, in parallel with transwell assays in the presence and absence of Matrigel (Fig. 2c, d). During the first 24 h, the time period in which the transwell assays were performed, neither the miR-181a-5p mimic nor the inhibitor had any obvious effect on cell number, compared to their respective negative controls, indicating that the difference in the number of invaded/migrated cells after miR-181a-5p mimic or inhibitor transfection was a primary effect of changes in the cell invasion and migration capacities, not due to secondary effects of cell proliferation. In addition, the ectopic-expression and inhibition of miR-181a-5p were confirmed by qRT-PCR after transfection (Fig. 2e, f). Collectively, these results indicated that miR-181a-5p suppresses HTR-8/SVneo cell invasion and migration.

### miR-181a-5p directly inhibits IGF2BP2

**Fig. 3:**
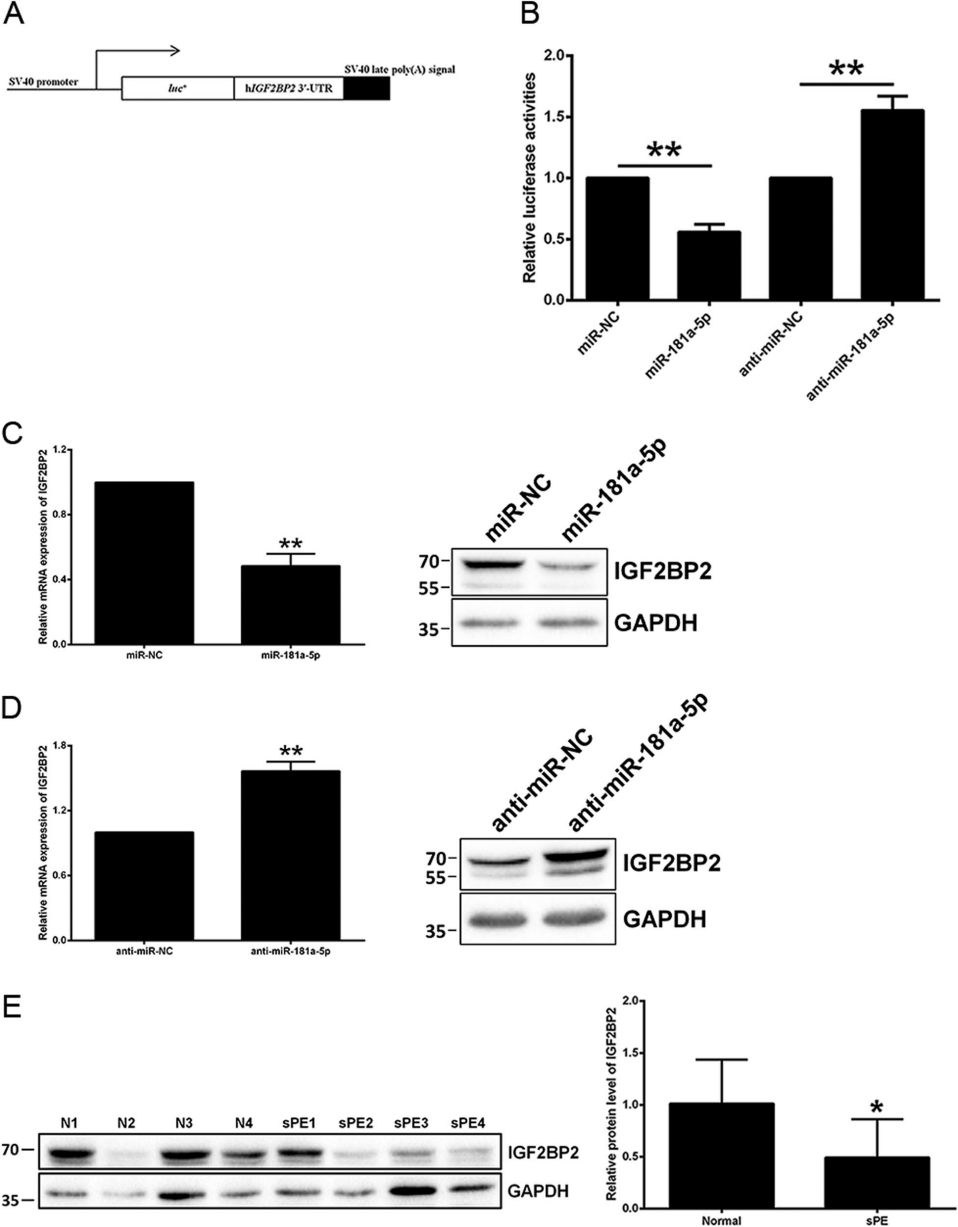
*IGF2BP2* is directly inhibited by miR-181a-5p **a** Construction of a pGL3-Control luciferase vector containing the full-length *IGF2BP2* 3ʹ-UTR. **b** The effects of miR-181a-5p mimic and inhibitor on the luciferase activity of the *IGF2BP2* WT 3ʹ-UTR reporter were measured. **c** The *IGF2BP2* mRNA and protein levels were both diminished by miR-181a-5p overexpression in HTR-8/SVneo cells. A representative western blotting image with the molecular weight markers depicted on the left in kDa is shown. **d** The *IGF2BP2* mRNA and protein levels were both elevated upon treatment of the miR-181a-5p inhibitor in HTR-8/SVneo cells. A representative western blotting image with the molecular weight markers depicted on the left in kDa is shown. **e** *IGF2BP2* protein level was assessed by western blotting in the 10 paired severe pre-eclamptic placentas and normal placentas mentioned in Fig. 1a. A representative western blotting image of four paired placentas is shown, and the molecular weight markers are depicted on the left in kDa. *IGF2BP2* protein level was statistically analyzed by quantitating the intensity of the IGF2BP2 bands relative to that of the corresponding GAPDH ones. *N* normal pregnancy, *sPE* severe pre-eclampsia. The results are expressed as the mean ± SD based on at least three independent experiments. *P < 0.05; **P < 0.01

To investigate the mechanism involved in miR-181a-5p suppressing the invasion and migration of trophoblasts, four computational algorithms, namely, TargetScan 7.0, PITA, PicTar, and miRanda were utilized to predict miR-181a-5p direct target genes. *IGF2BP2* was selected as a candidate of miR-181a-5p targets for further evaluation. To examine whether *IGF2BP2* is directly inhibited by miR-181a-5p, its full-length 3ʹ-UTR was introduced into the pGL3-Control luciferase vector (Fig. 3a). After co-transfection with miR-181a-5p mimic, the luciferase reporter activity was significantly decreased, indicating that miR-181a-5p directly inhibited *IGF2BP2*. Moreover, inhibition of endogenous miR-181a-5p by co-transfection with inhibitor of miR-181a-5p significantly increased luciferase reporter activity (Fig. 3b).

MiRNAs bind to target gene mRNAs for either mRNA degradation or translation repression^4^. To investigate how miR-181a-5p modulates *IGF2BP2* expression, we tested effects of miR-181a-5p on *IGF2BP2* mRNA/protein levels in HTR-8/SVneo cells. *IGF2BP2* mRNA levels declined by approximately one half after ectopically expressing miR-181a-5p (Fig. 3c). Consistent with this, a significant decrease of the endogenous *IGF2BP2* protein levels was caused by miR-181a-5p (Fig. 3c). Conversely, treatment with the miR-181a-5p inhibitor raised both mRNA/protein levels of *IGF2BP2* (Fig. 3d).

To evaluate whether placental expression of *IGF2BP2* is correlated to miR-181a-5p expression in diseased states, we examined *IGF2BP2* expression in 10 severe pre-eclamptic placentas and 10 normal placentas by western blotting. Of interest, severe pre-eclamptic placentas showed significantly higher miR-181a-5p level (Fig. 1a) and significantly lower *IGF2BP2* expression (Fig. 3e). The same inverse correlation between the expression levels of *IGF2BP2* and miR-181a-5p was also evident in the three trophoblast cell lines: High *IGF2BP2*-expressing HTR-8/SVneo and JAR cells had relatively lower miR-181a-5p expression, while low *IGF2BP2*-expressing JEG-3 cells exhibited higher miR-181a-5p levels (Fig. 1b and Supplementary Fig. 1).

### miR-181a-5p-mediated repression of IGF2BP2 occurs via a conserved binding site in 3ʹ-UTR of IGF2BP2 mRNA

**Fig. 4:**
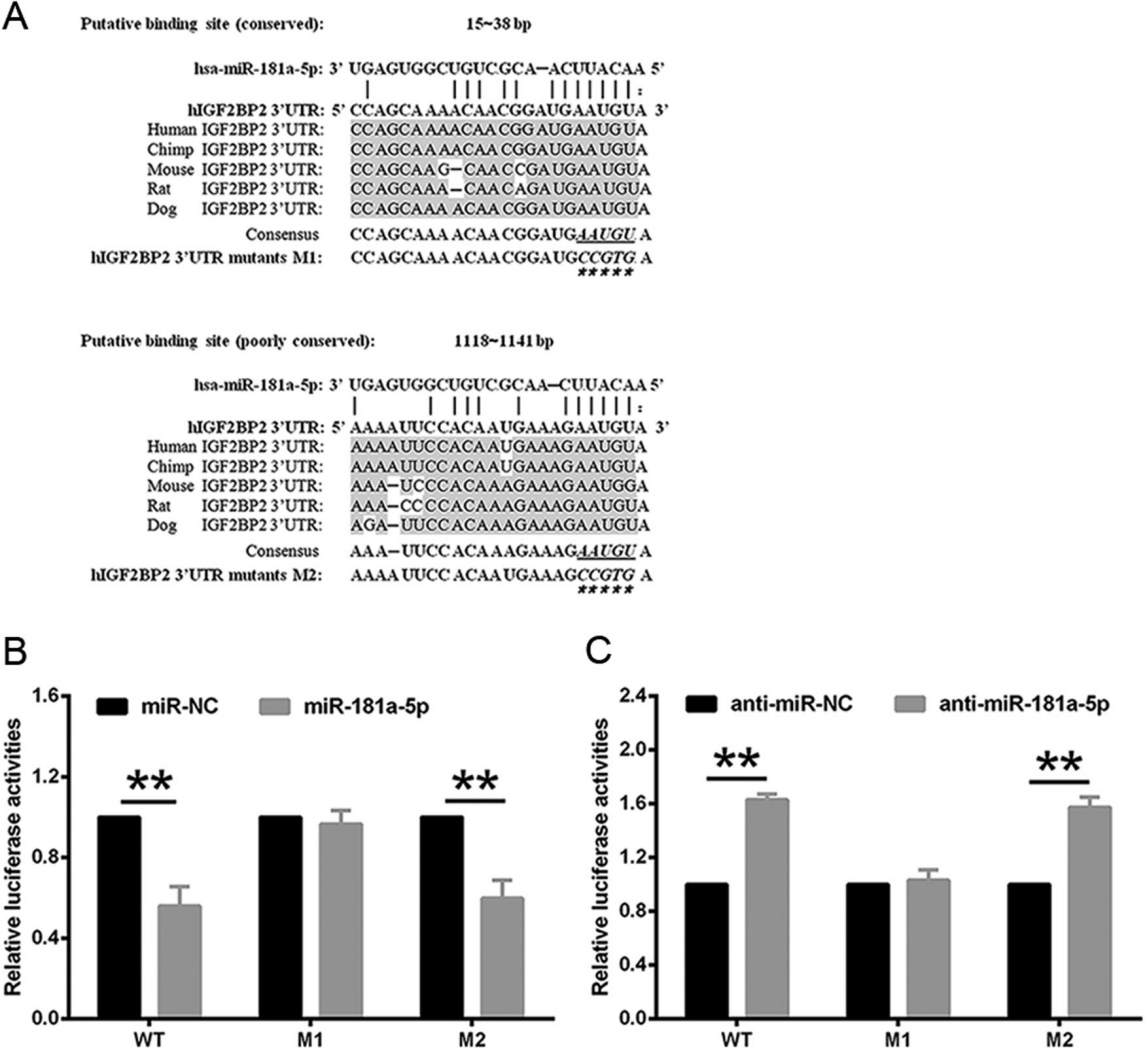
A miR-181a-5p binding site exists in the 3ʹ-UTR of *IGF2BP2* mRNA **a** Two putative miR-181a-5p binding sites are shown in the 3ʹ-UTR of *IGF2BP2* mRNA. **b**, **c** The luciferase activities of reporter vectors containing either the WT or the M1/M2 mutant 3ʹ-UTR were measured in the presence of miR-181a-5p mimic or inhibitor. The results are expressed as the mean ± SD based on at least three independent experiments. **P < 0.01

MiRNAs work by first binding to mRNA via specific target sites that are typically evolutionarily conserved and perfectly matched to the 5ʹ end of the miRNAs^15^. Two predicted positions for miR-181a-5p binding to *IGF2BP2* mRNA were shown in the 3ʹ-UTR: one locates at 15–38 bp and is highly conserved across species, while the other locates at 1118–1141 bp and is poorly conserved (Fig. 4a). To determine whether the inhibition of *IGF2BP2* by miR-181a-5p occurred via these predicted miR-181a-5p-binding sites, the above two sites were mutated, respectively, and referred as the M1 and M2 mutants. Luciferase reporter assays indicated that the M1 mutant 3ʹ-UTR interrupted miR-181a-5p-mediated repression, whereas the M2 mutant 3ʹ-UTR showed similar inhibition as wild-type (WT) 3ʹ-UTR when treated with the miR-181a-5p mimic (Fig. 4b). Consistent with this, inhibiting endogenous miR-181a-5p by its inhibitor raised the activities of the WT and M2 mutant 3ʹ-UTR reporter but not that of the reporter containing M1 mutant 3ʹ-UTR (Fig. 4c). These results suggested that the highly conserved sequence locating at 15–38 bp of the *IGF2BP2* 3ʹ-UTR is the major miR-181a-5p binding site, and further confirmed that miR-181a-5p directly inhibits *IGF2BP2*.

### siRNAs targeting IGF2BP2 imitate the effects of overexpressed miR-181a-5p on trophoblast cell invasion and migration

**Fig. 5:**
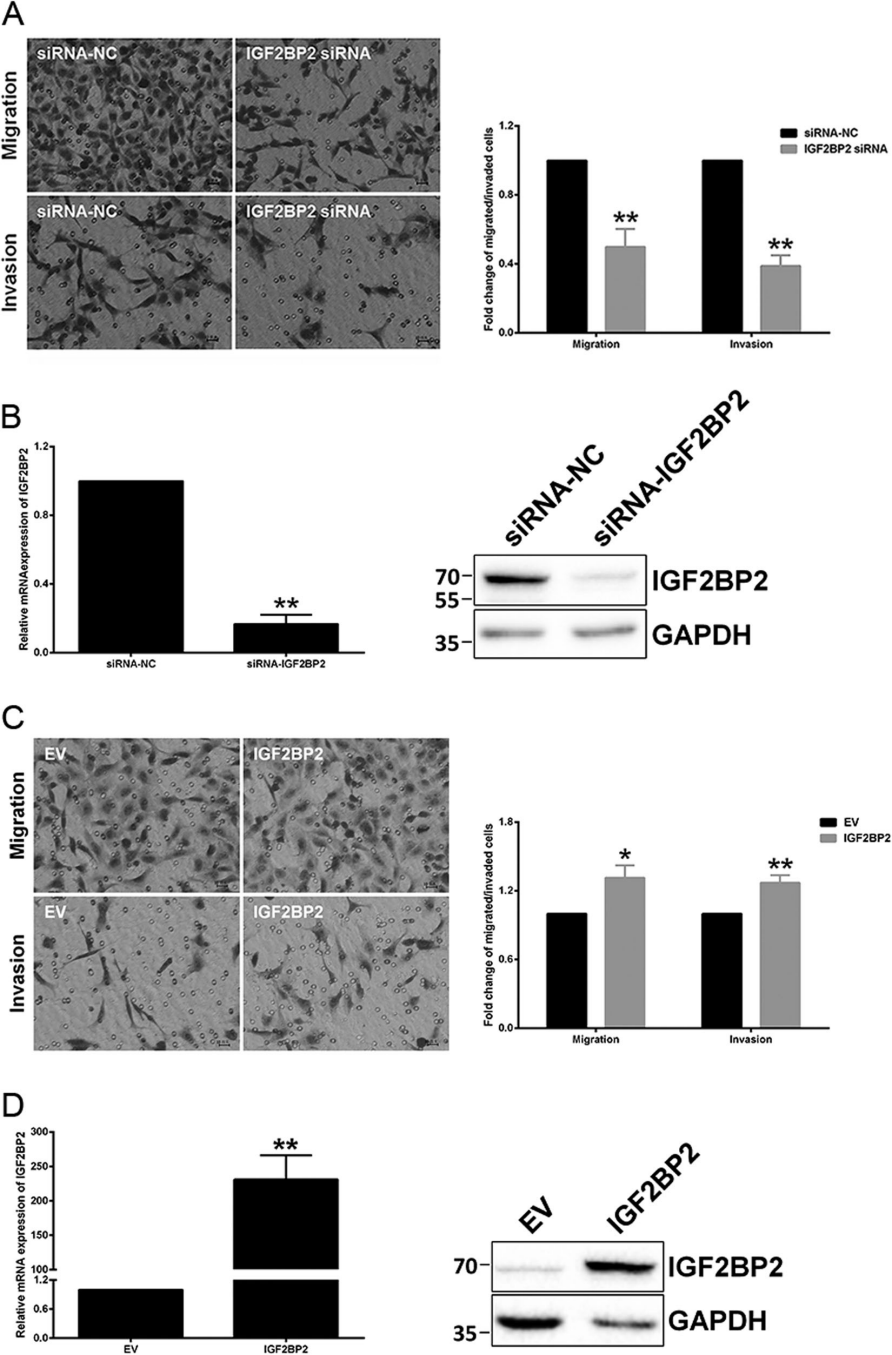
siRNAs targeting *IGF2BP2* imitate the effects of overexpressed miR-181a-5p on HTR-8/SVneo cell invasion and migration **a** Transfection of *IGF2BP2* siRNA significantly reduced HTR-8/SVneo cell invasion and migration, with effects similar to those of miR-181a-5p overexpression. Representative fields of invaded/migrated cells (at 200× original magnification, bar = 10 μm) are shown. **b** The *IGF2BP2* mRNA/protein levels were examined after siRNA transfection. A representative western blotting image with the molecular weight markers depicted on the left in kDa is shown. **c** Ectopic-expression of *IGF2BP2* significantly promoted HTR-8/SVneo cell invasion and migration. Representative fields of invaded/migrated cells (at 200× original magnification, bar = 10 μm) are shown. **d** The *IGF2BP2* mRNA/protein levels were examined after plasmid transfection. A representative western blotting image with the molecular weight markers depicted on the left in kDa is shown. The results are expressed as the mean ± SD based on at least three independent experiments. *P < 0.05; **P < 0.01

*IGF2BP2* siRNA was introduced into HTR-8/SVneo cells to study whether the effects on trophoblast invasion and migration by miR-181a-5p were mediated via its direct target, *IGF2BP2*. As expected, cell invasion and migration were significantly reduced upon transfection of *IGF2BP2* siRNA, with effects similar to those after miR-181a-5p overexpression (Fig. 5a). *IGF2BP2* expression levels were examined in parallel after siRNA transfection (Fig. 5b). The suppressive effects of *IGF2BP2* siRNA on trophoblast invasion and migration was further investigated in JEG-3 cells with miR-181a-5p inhibition. Excitingly, transfection of *IGF2BP2* siRNA not only resulted in an inhibition of JEG-3 cell invasion and migration (Supplementary Fig. 2a), but also abolished the invasion/migration-stimulative effects of miR-181a-5p inhibitor on JEG-3 cells (Supplementary Fig. 2c). IGF2BP2 expression levels were examined in parallel (Supplementary Fig. 2b and d). Conversely, ectopic-expression of *IGF2BP2* significantly promoted invasion and migration of HTR-8/SVneo (Fig. 5c) and JAR cells (Supplementary Fig. 3a). IGF2BP2 expression levels were examined in parallel in HTR-8/SVneo (Fig. 5d) and JAR cells (Supplementary Fig. 3b).

### miR-181a-5p suppresses trophoblast invasion and migration via directly inhibiting IGF2BP2

**Fig. 6:**
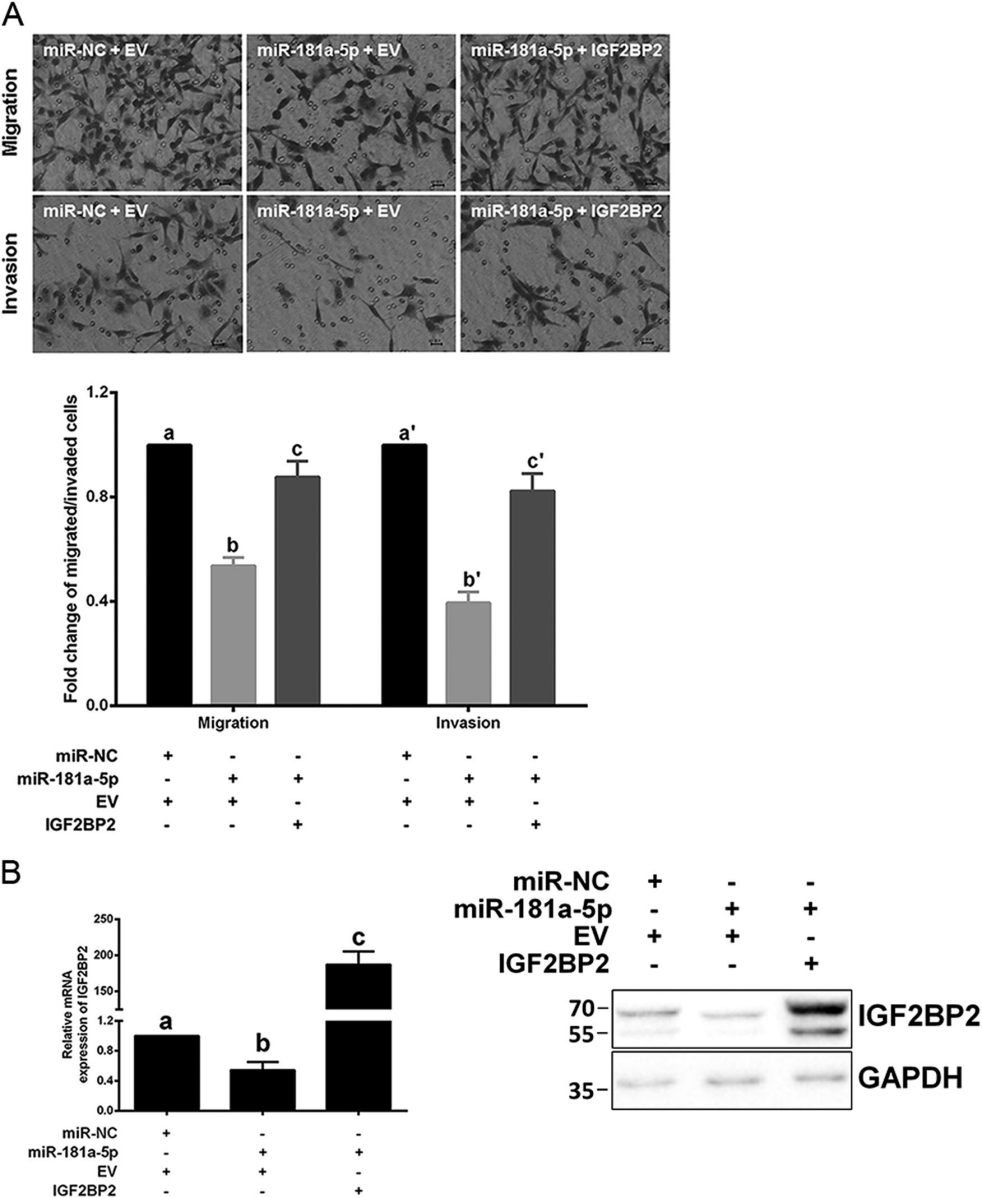
miR-181a-5p suppresses HTR-8/SVneo cell invasion and migration via directly inhibiting IGF2BP2 **a** Restoring *IGF2BP2* expression partially reversed the inhibitory effects of miR-181a-5p on HTR-8/SVneo cell invasion and migration. Representative fields of invaded/migrated cells (at 200× original magnification, bar = 10 μm) are shown. **b** The *IGF2BP2* mRNA/protein levels were examined after *IGF2BP2* restoration. A representative western blotting image with the molecular weight markers depicted on the left in kDa is shown. The results are expressed as the mean ± SD based on at least three independent experiments. The values with diverse letters are significantly different (*P* < 0.05)

To validate that the suppressive function of miR-181a-5p on trophoblast invasion and migration were mediated by *IGF2BP2* repression, *IGF2BP2* expression was rescued by co-overexpression with miR-181a-5p in HTR-8/SVneo cells. Excitingly, restoring *IGF2BP2* expression partially reversed the inhibitory effects of miR-181a-5p on HTR-8/SVneo cell invasion and migration (Fig. 6a). *IGF2BP2* expression levels were examined in parallel after co-transfection (Fig. 6b). Consistent with this, restoring *IGF2BP2* expression also partially rescued the inhibitory effects on JAR cell invasion and migration by miR-181a-5p (Supplementary Fig. 3c). The *IGF2BP2* protein levels were examined in parallel after co-transfection (Supplementary Fig. 3d). These results suggested that miR-181a-5p suppresses trophoblast invasion and migration at least partially by directly inhibiting *IGF2BP2*.

## Discussion

In eutherian organisms, the placenta interfaces the fetal and maternal environments and is obligatory for supporting fetal development and growth; dysregulated placental development leads to diseases of pregnancy, such as PE, as maternally accompanied with hypertension, proteinuria, and systemic vasculopathy that impacts many maternal organs, and with a secondary effect on fetal growth and health^9^. MiRNAs are intimately involved in human development and disease^16^. The discovery of dysregulated miRNAs, e.g., miR-210, miR-376c, and miR-455, and their gene-regulatory roles in placental development has provided a new avenue for elucidating the underlying mechanisms of pregnancy-specific diseases, such as PE^17–19^. In the present work, we demonstrated that miR-181a-5p, a miRNA that is increased in both the plasma and placenta of severe pre-eclamptic patients compared to those experiencing normal pregnancies, inhibits trophoblast invasion and migration by directly targeting *IGF2BP2*.

Up-regulation of miR-181a-5p in circulation was first found in patients with sPE in our previous study^10^. Several other studies indicated that this increase of miR-181a-5p also exists in placenta from severe pre-eclamptic patients^7,8,12^, which was confirmed in the present study. MiR-181a-5p has been extensively studied and reported to have essential roles in T cell sensitivity and selection^20–22^, multiple myeloma pathogenesis^23–26^, radio/chemo-therapeutic resistance of cancer^27–29^, stroke^30,31^, and autophagy^32,33^. In reproductive systems, miR-181a-5p was reported to mediate the effects of anti-Müllerian hormone (AMH) on follicular development^34^. In addition, miR-181a-5p was significantly decreased in seminal plasma from azoospermia but increased in that from asthenozoospermia compared to age-matched fertile controls^35^. However, whether miR-181a-5p has roles in trophoblast differentiation and placental development and whether its dysregulation contributes to PE development remain unknown.

It is widely accepted that trophoblast differentiation in the pre-eclamptic placenta may be abnormal as early as the first trimester, which is long before the clinical manifestations of PE can be detected, leading to incomplete vascular remodeling caused by impaired deep trophoblast invasion^13,36^. In this work, we first determined miR-181a-5p expression in term-matched normal placentas and severe pre-eclamptic placentas and in three trophoblasts lines. HTR-8/SVneo cells, a extravillous trophoblasts line established by stably transfecting normal human first trimester trophoblasts with the gene encoding SV40 large T-antigen^14^, were primarily utilized to study the roles of miR-181a-5p in trophoblast invasion/migration. Transfection with either mimic or inhibitor of miR-181a-5p significantly suppressed or enhanced, respectively, HTR-8/SVneo cell invasion and migration.

One bioinformatic prediction of *IGF2BP2* directly inhibited by miR-181a-5p was validated by using luciferase reporter assays and qRT-PCR/western blotting. Furthermore, a conserved miR-181a-5p binding site in the 3ʹ-UTR of *IGF2BP2* mRNA was identified by sites-mutagenesis. In addition, siRNAs targeting *IGF2BP2* imitated the miR-181a-5p ectopic-expression in suppressing HTR-8/SVneo cell invasion and migration, whereas restoring *IGF2BP2* expression with an *IGF2BP2*-coding plasmid partially rescued the inhibitory abilities of miR-181a-5p in HTR-8/SVneo cell invasion and migration. These results suggested that miR-181a-5p suppresses cytotrophoblast invasion and migration at least partially by directly targeting *IGF2BP2*.

MiR-181a-5p plays critical roles in invasion/migration of various cancer types: it promotes invasion and migration in ovarian cancer^37^ and prostate cancer^38^ but inhibits invasion and migration in breast cancer^39^; this may be due to the existence of tissue-specific targets. *IGF2BP2* is one member of the *IGF2BP* family that has previously been regarded as oncofetal, as its members were originally discovered in developing embryos^40^. *IGF2BP2* is an important gene associated with type 2 diabetes, and a recent study found that *Igf2bp2*^−/−^ mice were lean and resistant to diet-induced obesity^41^. In addition, *IGF2BP2* plays critical roles in myogenesis^42,43^, autophagy transition^44^, and the promotion of cell migration and invasion in cancer^45,46^. However, *IGF2BP2* has never been studied in trophoblast invasion and migration, although another *IGF2BP* family member, *IGF2BP3*, was found to stimulate invasion and migration of trophoblasts^47^. In our present work, *IGF2BP2* siRNA impaired invasion and migration of HTR-8/SVneo cells, and *IGF2BP2* overexpression rescued the suppressive effects of miR-181a-5p on HTR-8/SVneo cell invasion and migration, suggesting an important role for *IGF2BP2* in trophoblast invasion and migration that has never been reported before. Interestingly, we also demonstrated the decreased *IGF2BP2* expression in severe pre-eclamptic placentas that expressed increased miR-181a-5p.

In summary, we demonstrated that miR-181a-5p, a miRNA elevated in both the plasma and placenta of severe pre-eclamptic patients, suppresses trophoblast invasion and migration by directly targeting *IGF2BP2*. However, one limitation is that the data presented in this study were mainly obtained from human trophoblast cell lines in vitro. The “in vivo” function of miR-181a-5p in placental development and in the pathogenesis of sPE need to be further explored, for example, in mouse with miR-181a-5p conditionally deleted in its placenta. Another limitation of this study is that we were unable to test whether differential miR-181a-5p expression exists between primary human trophoblast cells from pregnant females who would or would not develop clinical manifestations of sPE at the first trimester (when trophoblast differentiation occurs). Further work is needed to identify circulating miRNAs, such as miR-181a-5p, that could serve as biomarkers for the non-invasive predictive diagnosis of sPE as early as the first trimester.

## Materials and methods

### Sample collection

For the confirmation of reported placental miR-181a-5p elevation in sPE^7,8,12^, term-matched placenta samples between 36 and 40 weeks were obtained with informed consent from women experiencing severe pre-eclamptic pregnancies (sPE group; *n* = 10) and normal pregnancies (normal group; *n* = 10) at the Department of Gynecology and Obstetrics in the First Affiliated Hospital of Zhengzhou University. Severe pre-eclamptic pregnancies were recruited according to the definition in Williams Obstetrics (23rd edition). Briefly, these pregnant patients, with no history of pre-existing/chronic hypertension, exhibited either pressure of systolic blood ⩾ 160 mm Hg or pressure of diastolic blood ⩾110 mm Hg by ⩾ 2 points (⩾6 h apart) when patients rested on bed, accompanied by severe proteinuria (urinary protein excretion, ⩾2 g/24 h) after the first 20 weeks of gestation. No other maternal complications presented in the sPE pregnancies. The patient characteristics of these two groups are summed up (Table 1), and the research protocols were approved via the Ethics Committee of the First Affiliated Hospital of Zhengzhou University. All pregnancies were treated by elective cesarean delivery in the absence of labor. Within 1 h of cesarean birth, four tissue blocks (~0.3 cm^3^ each) were sampled randomly around the position of umbilical cord insertion site at the decidual side of each placenta to achieve adequate and uniform sampling and instantly snap-frozen/stored with liquid nitrogen.

**Table 1:** Clinical characteristics of the pregnant women participated in this study

**Characteristics**	**Normal** (*n* = 10)	**sPE** (*n* = 10)	***P***-**value**
Maternal age (years)	29.3 ± 2.7	29.7 ± 3.1	0.762
Pre-pregnancy body mass index (kg/m^2^)	21.4 ± 2.3	22.5 ± 2.3	0.335
Systolic blood pressure (mmHg)	114.6 ± 10.7	160.4 ± 19.5^a^	<0.001
Diastolic blood pressure (mmHg)	73.7 ± 8.0	100.7 ± 12.1^a^	<0.001
Proteinuria (g/24 h)	Normal/non-detected	3.7 ± 1.2^a^	<0.001
Primiparae (*n*)	6 (60%)	5 (50%)	NA
Current smoker (*n*)	0 (0%)	0 (0%)	NA
Han ethnicity (*n*)	10 (100%)	10 (100%)	NA
Female fetus (*n*)	5 (50%)	5 (50%)	NA
Gestational age at delivery (weeks)	37.7 ± 1.3	36.8 ± 0.4	0.051
Birth weight (g)	3165 ± 440.7	2304 ± 274.8^a^	< 0.001

Values are expressed as the mean ± SD, and statistical analyses were performed by using one-way ANOVA.

*sPE *severe pre-eclampsia, *NA* not analyzed

^a^Compared to normal pregnancy, *P* < 0.01

### Cell lines and cell culture

The human extravillous trophoblasts line HTR-8/SVneo was generously provided by Professor C. H. Graham at Queen’s University, Canada and was cultured in Gibco RPMI 1640 medium (Life Technologies, Carlsbad, CA, USA) containing 10% Gibco fetal bovine serum (FBS), 100 units/ml penicillin and 100 μg/ml streptomycin. The choriocarcinoma cell line JEG-3 (provided by ATCC in USA) was a kind gift from Professor Yanling Wang at the Institute of Zoology, Chinese Academy of Sciences, Beijing, China and was cultured in Gibco high-glucose DMEM medium with 10% FBS and penicillin (100 units/ml)/streptomycin (100 μg/ml). The choriocarcinoma cell line JAR was provided by the Cell Bank of Chinese Academy of Sciences in Shanghai, China with authentication using short tandem repeat DNA profiling and test for mycoplasma contamination using Hoechst DNA staining, and cultured in Gibco RPMI 1640 medium, with the same addition of FBS and antibiotics as the medium for HTR-8/SVneo cell culture. Cells were incubated at 37 °C with 5% CO_2_ and routinely passaged every 3 days. All the three cell lines used in this study are not listed in the database of commonly misidentified cell lines maintained by ICLAC and NCBI Biosample.

### Plasmid construction

The full-length (1792 base pairs (bp)) 3ʹ-UTR of *IGF2BP2* was cloned from HeLa genomic DNA and inserted into pGL3-Control luciferase vector (Promega, Madison, WI, USA) following instructions from manufacture. The *IGF2BP2* 3ʹ-UTR mutant vectors, with the first five nucleotides of the sequence complemented to the seed positions of miR-181a-5p were changed, were generated using the Gibson Assembly Cloning Kit (NEB, Ipswich, MA, USA). The full-length (1797 bp) *IGF2BP2* CDS lacking the start codon was generated by RT-PCR using total RNA extracted from HTR-8/SVneo cells and inserted into the *EcoR* I and *Bgl* II sites downstream of the FLAG peptide sequence in the N-terminal pFLAG-CMV-4 vector (Sigma, St. Louis, MO, USA). An empty N-terminal pFLAG-CMV-4 vector was served as a negative control. The pRenilla-TK vector, an internal control of the dual-luciferase assay, was a generous gift from Professor Qiang Wang at the Institute of Zoology, Chinese Academy of Sciences, Beijing, China.

### Oligonucleotide and plasmid transfection

Hsa-miR-181a-5p mimic and inhibitor, siRNAs targeting *IGF2BP2*, and their corresponding negative controls were obtained from Life Technologies (mirVana™/Stealth RNAi™). Cells were seeded in 35 mm dishes with growth medium and antibiotics 18 h before transfection. Transient transfections were performed using Lipofectamine RNAiMAX (for oligonucleotide transfections) or Lipofectamine 2000 (for plasmid transfections) from Life Technologies when cells were 50–60% confluent and oligonucleotide–lipid or plasmid–lipid mixture was prepared by following the instruction from manufacturer. Cells were obtained 2–3 days after transfection for further investigation.

### In vitro invasion/migration assays

Invasion assays were conducted in transwell inserts (Costar, Cambridge, MA, USA) pre-coated by Matrigel (BD Biosciences, Beit-Ha’ Emek, Israel) as previously reported^48^. In brief, HTR-8/SVneo and JAR cells (0.7 × 10^5^ cells for the assays with single ectopic-expression of *IGF2BP2*, and 1.5 × 10^5^ cells for the other assays) suspended in 200 μl RPMI 1640 medium without FBS or JEG-3 cells (3 × 10^5^ cells) suspended in 200 μl high-glucose DMEM medium in the absence of FBS were laid in the Matrigel (50 μl of 1 mg/ml)-coated upper compartment of transwell inserts, whereas the lower well contained corresponding mediums (600 μl with 10% FBS). After 22 h 37 °C incubation with 5% CO_2_, non-invaded cells attaching to the top side of the insert membrane were cleared by cotton swabs. The cells invading to the bottom side of the insert membrane were gently cleaned by PBS and immediately immersed in pre-chilled methanol (−20 °C) for 12-min fixation. Fixed cells were subsequently incubated with hematoxylin for 12-min staining. Stained cells were photographed of five randomly selected non-overlapped fields visualized at 200 × magnification by using a DMI6000 B microscope (Leica, Heidelberg, Germany) equipped with a DFC420 camera (Leica), and counted for the estimation of cell invasion. All the images were processed with Photoshop CS6 (Adobe Systems, San Jose, CA, USA). The number of invaded cells with different treatments was normalized to the corresponding controls. The results are expressed as the mean ± SD, and representative fields of invaded cells (at 200 × original magnification) are shown.

The migration assays were performed similarly to the invasion assays, except the inserts were not conducted with pre-coating of Matrigel. The number of migrated cells with different treatments was also normalized to the corresponding controls. The results are expressed as the mean ± SD, and representative fields of migrated cells (at 200× original magnification) are shown.

### Cell counting Kit-8 (CCK-8) assay

Two days after transfection, 5000 cells were laid per well of 96-well plates (five replicate wells for each condition). CCK-8 assays were performed at 0 (plated with medium containing CCK-8), 24, 48, and 72 h after cell-plating. Culture medium was changed every 2 days, if necessary. For each assay, culture medium was changed to 100 µl of CCK-8 medium (DOJINDO, Japan) and kept for further 2 h. The plates were read on a Varioskan™ Flash Microplate Reader (Thermo Scientific, Waltham, MA, USA) at wavelength of 450 nm after 2-h incubation and the mean absorbance of five replicate wells per condition was recorded.

### qRT-PCR

Extraction of total RNA was performed with TRIzol reagent (Life Technologies) following the manufacturer’s instruction; extracted RNA was quantified with NanoDrop 2000c (Thermo Scientific). Stem-loop qRT-PCR for miRNA quantification was conducted as reported in our previous work^10^. One microgram of total RNA was used for each stem-loop RT reaction. Expression of miR-181a-5p was normalized to the small nuclear RNA U6 expression as an endogenous control. *IGF2BP2* expression was examined via standard qRT-PCR and was normalized to glyceraldehyde-3-phosphate dehydrogenase (*GAPDH*) expression as an endogenous control. All the primers were ordered from Invitrogen, Beijing, China, and the sequences are summed up (Table 2).

**Table 2:** Primers used in qRT-PCR analysis

**Genes**	**Primers**	**Sequence**(5ʹ–3ʹ)
hsa-miR-181a-5p	RT	GTCGTATCCAGTGCAGGGTCCGAGGTATTCGCACTGGATACGACACTCAC
	PCR-F	GCCGAACATTCAACGCTGTCG
	PCR-R	GTGCAGGGTCCGAGGT
U6	RT	AACGCTTCACGAATTTGCGT
	PCR-F	CTCGCTTCGGCAGCACA
	PCR-R	AACGCTTCACGAATTTGCGT
*IGF2BP2*	PCR-F	GTTCCCGCATCATCACTCTTAT
	PCR-R	GAATCTCGCCAGCTGTTTGA
*GAPDH*	PCR-F	ATGGAAATCCCATCACCATCTT
	PCR-R	CGCCCCACTTGATTTTGG

*hsa* *Homo sapiens*, *F* forward, *R* reverse

### Target prediction

MiR-181a-5p target genes were predicted using four free databases, including TargetScan 7.0, PITA, PicTar, and miRanda, with the default parameters.

### Dual-luciferase assay

HTR-8/SVneo cells were co-transfected with *IGF2BP2* 3ʹ-UTR WT/Mutant vector and the internal control pRenilla-TK, together with mimic/inhibitor of hsa-miR-181a-5p or the corresponding negative controls. Cells were split 2 days after transfection; luciferase activities were immediately assessed by using the Dual-Luciferase Reporter Assay Kit (Promega) in a Varioskan™ Flash Microplate Reader with luminometric detection.

### Western blotting

Extraction of total cell protein was carried out with lysis buffer^48^ containing a Protease Inhibitor Cocktail (Sigma). Protein concentration was calculated by standard Bradford assays (Beyotime, Shanghai, China) that were read on a Varioskan™ Flash Microplate Reader at wavelength of 595 nm. 10% SDS-PAGE was prepared for the separation of 25 μg protein of each sample and separated proteins were subsequently electro-transferred onto a PVDF membrane (Merck Millipore, Darmstadt, Germany). After 1 h of 5% skim milk (Bio-Rad, Hercules, CA, USA) blocking in room temperature, the membrane was immersed overnight (4 °C) in primary antibody against human IGF2BP2 (1:2000; ab124930, Abcam, Cambridge, UK), and that against GAPDH (1:10,000; ab181603, Abcam). The secondary goat anti-rabbit antibody was conjugated with HRP (1:10,000; ab6721, Abcam). All antibodies were diluted in 1× TBST containing 5% skim milk. Specific immunoreactive bands of proteins were photographed by using Pierce ECL western blotting substrate (Life Technologies) and a ChemiDoc MP System (BioRad). The signal intensities of the bands were quantitated by using ImageJ (National Institute of Health in USA).

### Statistical analysis

All data are expressed as the mean ± standard deviation (SD) based on at least three independent experiments, and Student’s *t*-test (SPSS Statistics 17.0, Chicago, IL, USA) was adopted to estimate the significance of the differences caused by treatments relative to their corresponding controls. Statistical significance is indicated mainly by one asterisk for *P* values less than 0.05 or by two asterisks for *P* values less than 0.01.

### Data availability

The data that support the prediction of hsa-miR-181a-5p direct target genes in this study are available from TargetScan 7.0 (www.targetscan.org), PITA (http://genie.weizmann.ac.il/pubs/mir07/mir07_dyn_data.html), PicTar (http://pictar.mdc-berlin.de), and miRanda (http://www.microrna.org/microrna/home.do) with the default parameters.

## Electronic supplementary material

Supplemental figure 1

Supplemental figure 2

Supplemental figure 3

Supplemental figure legends
