# Supplementary figures and images for: miR-181a-5p suppresses invasion and migration of HTR-8/SVneo cells by directly targeting IGF2BP2
Source: Cell Death Dis. 2018 Jan 16;9(2):16. doi: 10.1038/s41419-017-0045-0 (PMC5833820; doi:10.1038/s41419-017-0045-0)

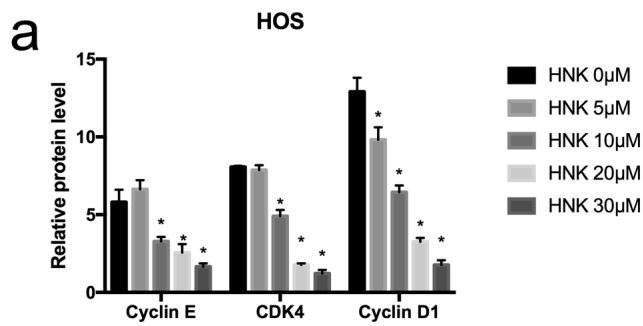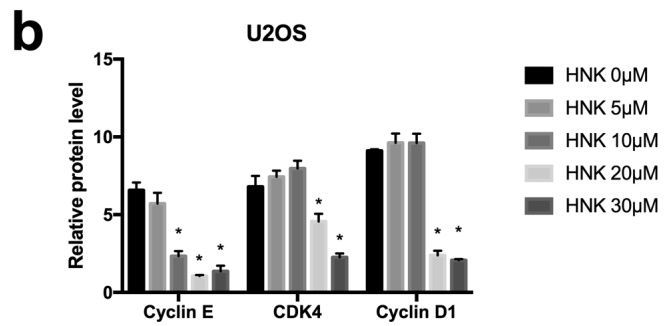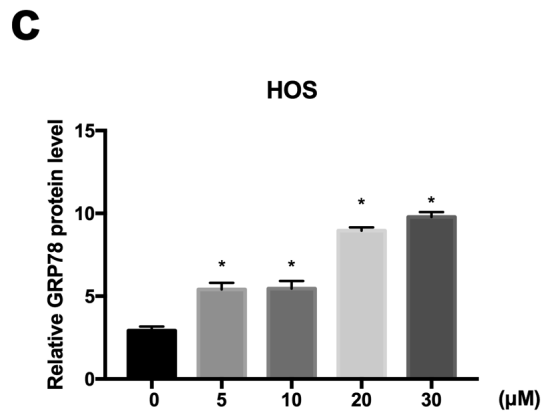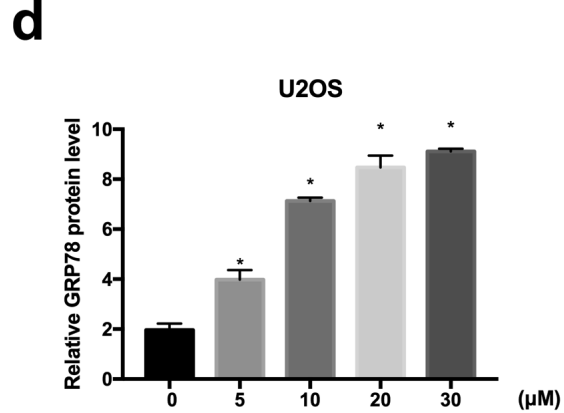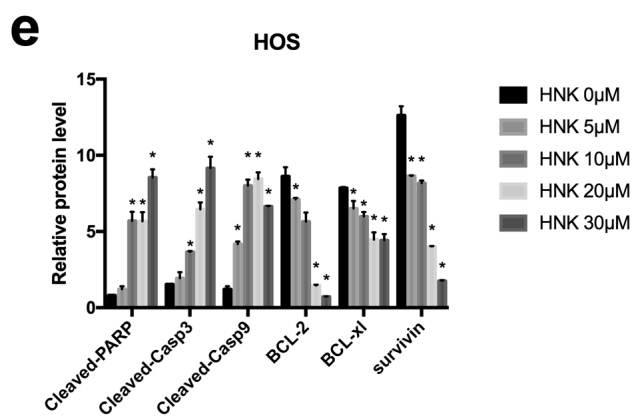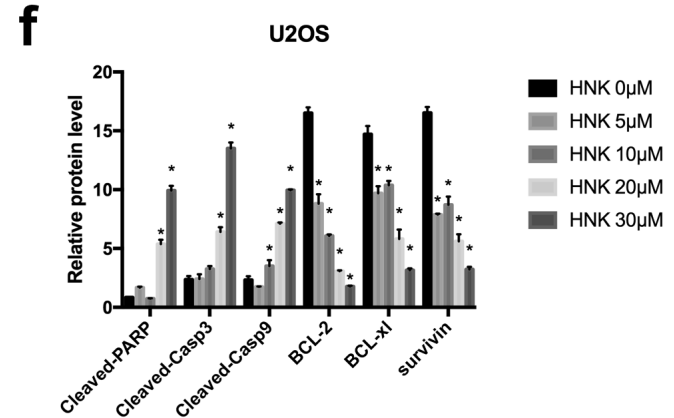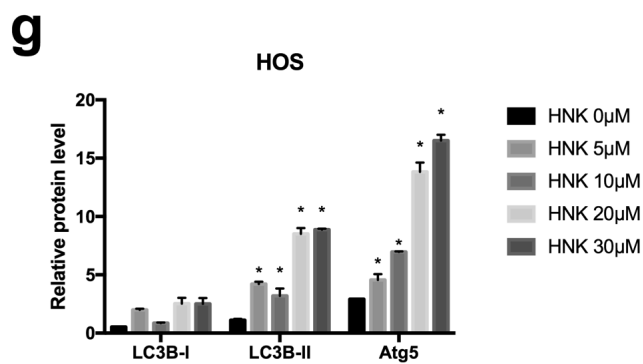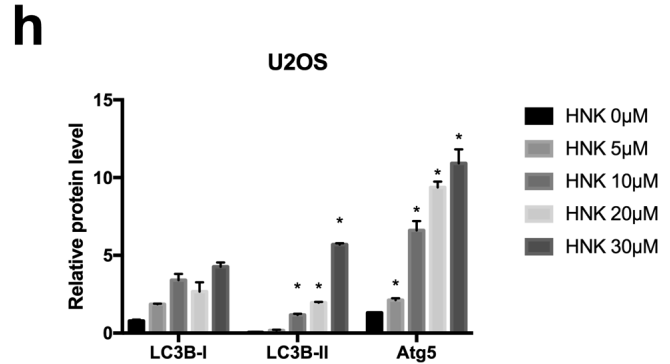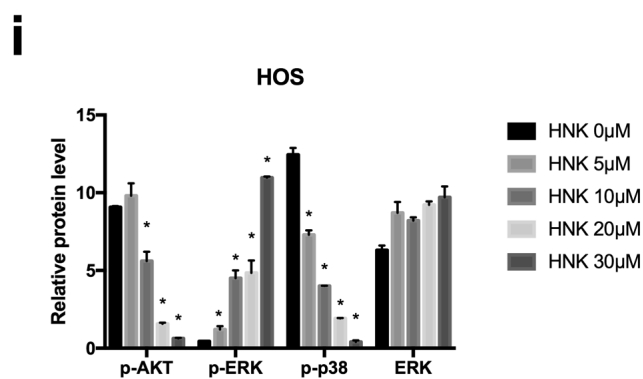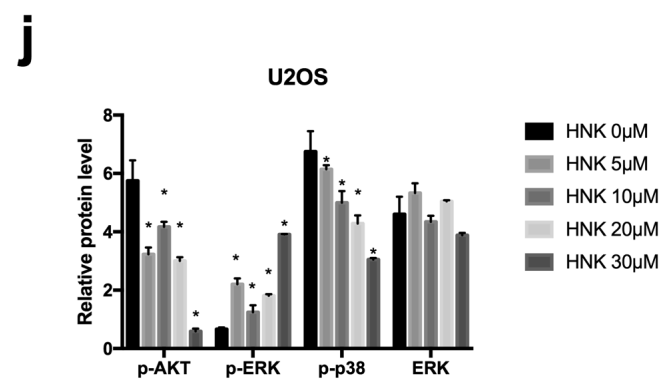

Supplement: Supplementary file 1 — Supplemental figure 1 [file 41419_2017_45_MOESM1_ESM.pdf]

a

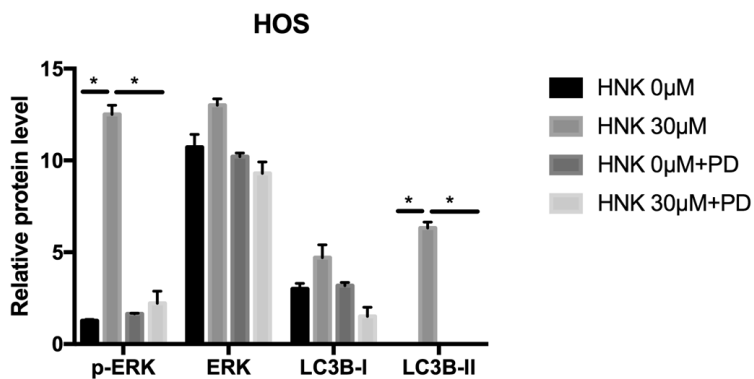

b

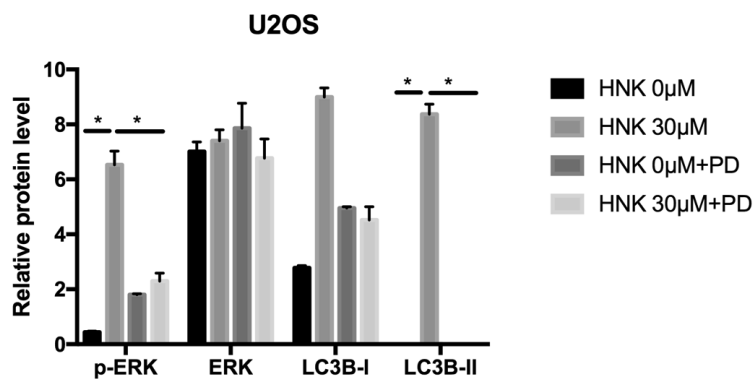

c

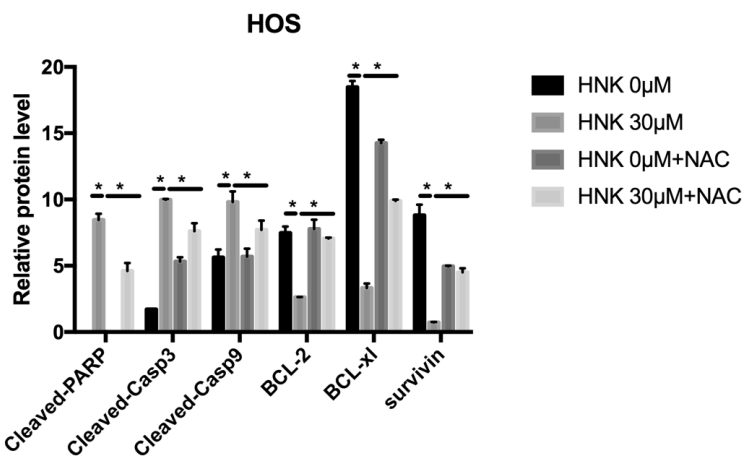

d

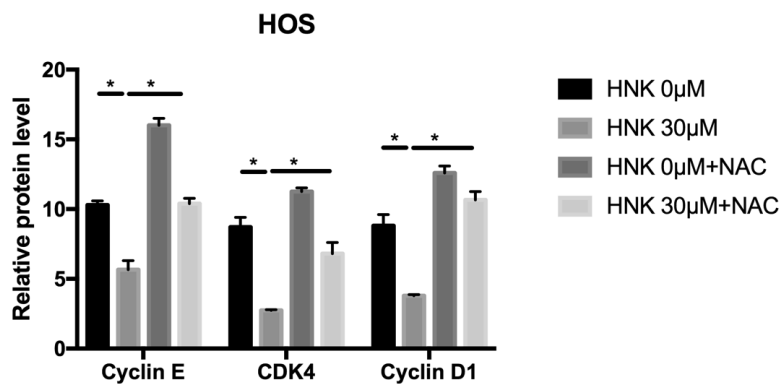

e

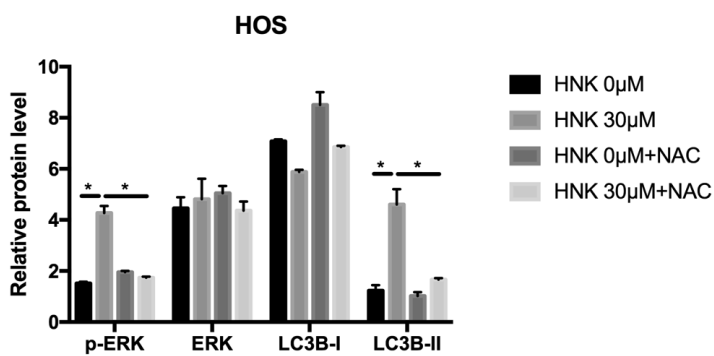

f

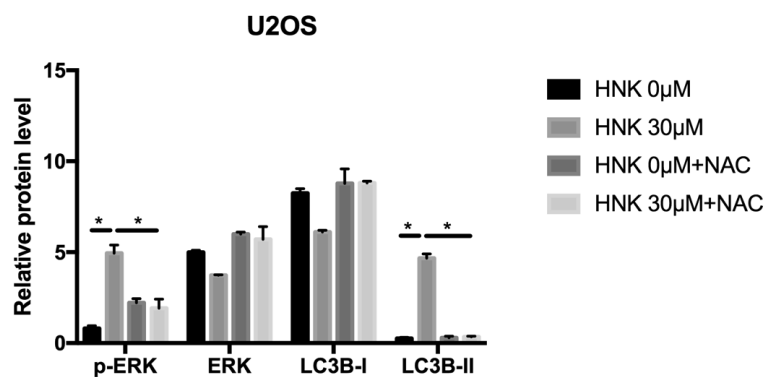

Supplement: Supplementary file 2 — Supplemental figure 2 [file 41419_2017_45_MOESM2_ESM.pdf]

**a**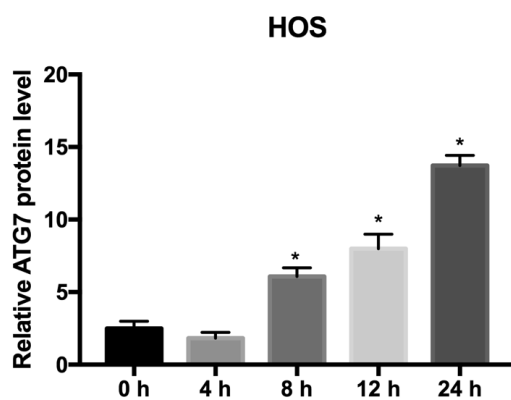**b**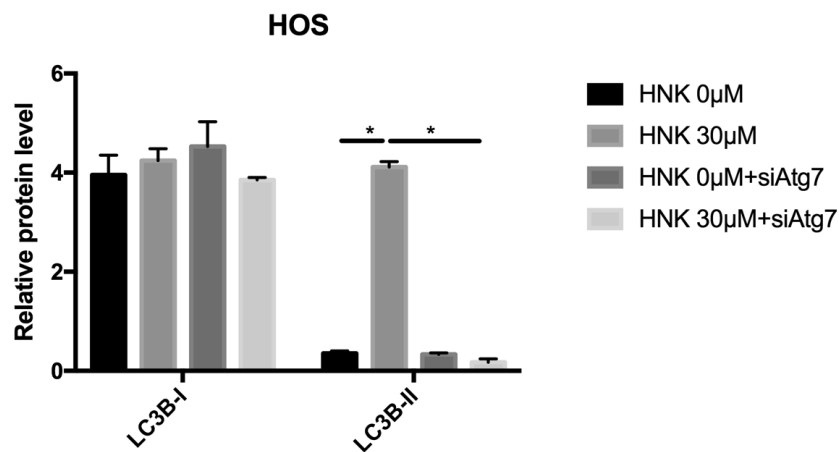**c**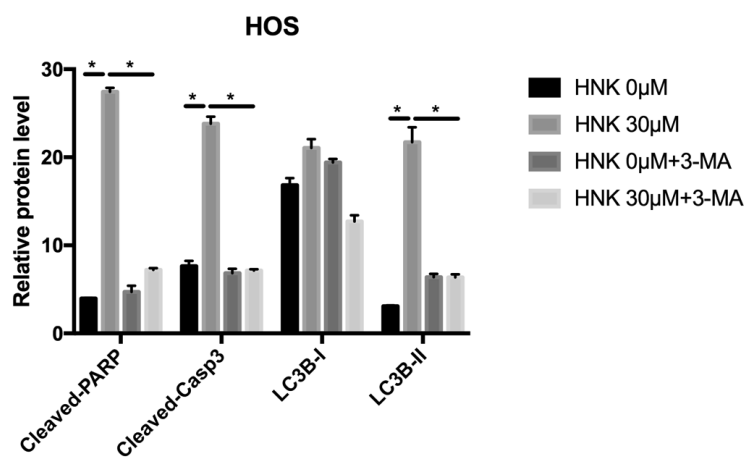**d**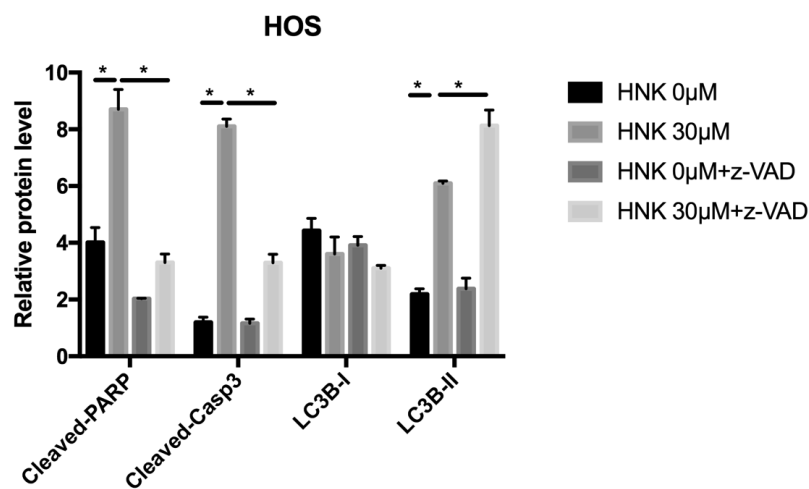**e**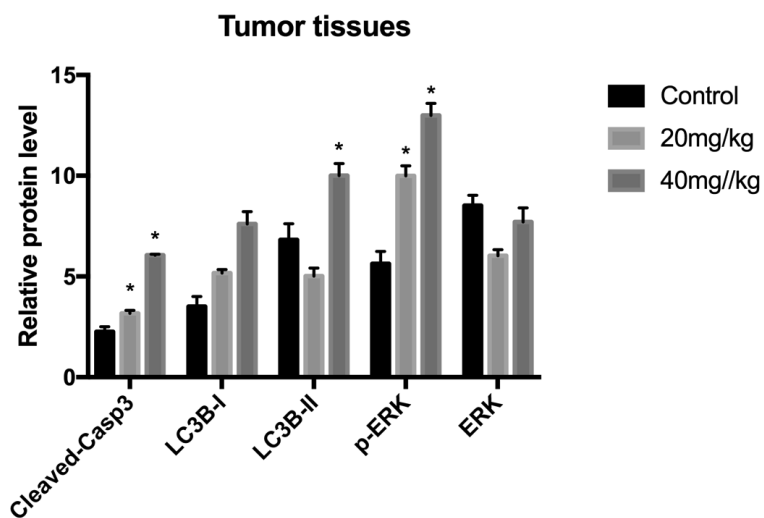

Supplement: Supplementary file 3 — Supplemental figure 3 [file 41419_2017_45_MOESM3_ESM.pdf]
